# Blood Pressure, Heart Rate, and CNS Stimulant Medication Use in Children with and without ADHD: Analysis of NHANES Data

**DOI:** 10.3389/fped.2014.00100

**Published:** 2014-09-19

**Authors:** Susan M. Hailpern, Brent M. Egan, Kimberly D. Lewis, Carol Wagner, Ghassan F. Shattat, Doaa I. Al Qaoud, Ibrahim F. Shatat

**Affiliations:** ^1^Independent Epidemiology Consultant, Saratoga, CA, USA; ^2^Greenville Health System, Care Coordination Institute, University of South Carolina School of Medicine, Greenville, SC, USA; ^3^College of Medicine, Medical University of South Carolina, Charleston, SC, USA; ^4^Neonatology, Medical University of South Carolina Children’s Hospital, Charleston, SC, USA; ^5^Faculty of Pharmacy, Al-Zaytoonah University of Jordan, Amman, Jordan; ^6^Pediatric Nephrology and Hypertension, Medical University of South Carolina Children’s Hospital, Charleston, SC, USA

**Keywords:** hypertension, heart rate, pediatrics, attention-deficit hyperactivity disorder, NHANES, central nervous system stimulants

## Abstract

It is estimated that 2–3% of children in the US have hypertension (HTN) and 8% of children ages 4–17 carry the diagnosis of attention-deficit hyperactivity disorder (ADHD). The prevalence of HTN and cardiovascular (CV) risk factors in children with ADHD on CNS stimulant treatment (stimulants) compared to no treatment and compared to their healthy counterparts is not well described. Using National Health and Nutrition Survey data, we examined demographic, blood pressure (BP) and CV risk factors of 4,907 children aged 12–18 years with and without the diagnosis of ADHD, and further examined the CV risk in a subgroup of ADHD patients on stimulants. Three hundred eighty-three (10.7%) children were reported to have ADHD, of whom 111 (3.4%) were on stimulants. Children with ADHD on stimulants were significantly younger, male, and white compared to those with ADHD not on medication and those without ADHD. Body mass index (BMI), eGFR, cholesterol, the prevalence of albuminuria, and poverty were not significantly different between the three groups. One hundred sixty (2.7%) had BP in the hypertensive and 637 (12.4%) in the pre-hypertensive range. The prevalence of elevated BP (HTN and/or pre-HTN range) was not different between children with ADHD on stimulants compared to ADHD without medication and those without ADHD. Heart rate (HR) was significantly higher in the ADHD group on stimulants vs. the groups ADHD on no stimulants and without ADHD. When the relationship between stimulants and the risk of abnormal BP was examined, there was a significant interaction between having BP in the HTN range and sex. After adjusting for BMI, race, and age, females with ADHD on stimulants tended to be older and had significantly more BP in the hypertensive range. On the other hand, males were more likely to be of a white race and older, but not hypertensive. Children with ADHD on stimulants have significantly higher HR than children with ADHD on no stimulants and children without ADHD. On the other hand, the prevalence of abnormal BP classification is comparable between the three groups.

## Introduction

Hypertension (HTN) in children and adolescents has been associated with target organ damage. It has been reported that as many as 34–38% of young adults with mild blood pressure (BP) elevation demonstrate left ventricular hypertrophy, which is the most common manifestation of target organ damage in childhood and adolescent HTN. Other associated comorbidities include retinopathy, cognitive impairment, and decreased glomerular filtration rate (GFR) and atherosclerotic plaques in the aorta and carotid arteries (1–4).

Centers for Diseases Control and Prevention (CDC) analysis of data from the 2007 National Survey of Children’s Health (NSCH) (5) found 9.5% of US children aged 4–17 years ever been diagnosed with attention-deficit hyperactivity disorder (ADHD), representing 5.4 million children in the US. Furthermore, ADHD diagnosis was two times more prevalent among males than females. Approximately 66.3% of children were reported to be taking medication for the disorder, with the highest prevalence of medication treatment among males aged 11–14 years. It should be noted that the prevalence of ADHD in children increased from 7.8% in 2003 to 9.8% in 2007 – a 21.8% increase in 4 years (5).

Stimulants are the mainstay of ADHD treatment. The effectiveness of stimulants in treating ADHD has been well documented. However, in 2006, because of concern over the cardiovascular (CV) effects of ADHD stimulants, the Drug Safety and Risk Management Advisory Committee of the Food and Drug Administration suggested a “black box” warning on stimulant drugs to treat ADHD (6).

The prevalence of HTN and other CV risk factors in children with ADHD on CNS stimulant medication compared to children with ADHD without CNS stimulant medication and children without ADHD is not well described. Using data from National Health and Nutrition Survey (NHANES) 1999–2004, we examined demographic and CV risk factors in children with and without the self-reported diagnosis of ADHD. In addition, we examined CV risk factors in children with ADHD on CNS stimulant medications. Finally, since differences in prevalence of ADHD, medication treatment of ADHD, and abnormal BP have been reported by sex, we tested a hypothesis of an interaction between BP and sex among children with ADHD on CNS stimulants.

## Materials and Methods

### Study population

Data from NHANES for the years 1999–2004 were analyzed. NHANES is an ongoing nationally representative cross-sectional survey of the civilian, non-institutionalized US population that uses a complex, stratified, and multistage probability design. The survey is performed by the National Center for Health Statistics at the CDC and released in 2-year increments. Survey participants underwent standardized interviews, physical examinations, and laboratory testing in their homes and at a mobile examination center (MEC). Height, weight, and BP measurements were obtained on all NHANES participants aged 12–18 years.

National Health and Nutrition Survey 1999–2004 was approved by the National Center for Health Statistics Institutional Review Board. All of the participants 18 years of age provided informed consent and for those participants under the age of 18 years, parents/guardians provided informed consent.

Participant use of CNS stimulants was obtained from self-report. A personal interview was conducted as part of NHANES protocol and participants were queried on use of prescription medication during a 1-month period prior to the survey date. Participants 16 years of age and younger had a proxy respond for them while those older than 16 years responded for themselves.

### Study variables

Demographic variables included in the current study are age (years), sex, and self-reported race/ethnicity (categorized as Mexican-American, Black, White, and other). Poverty–income-ratio (PIR) is a ratio of a family’s income to the poverty threshold as defined by the US Census Bureau. A PIR ≤1 was defined as below the poverty threshold by NHANES.

Physical examination measures were obtained in the MEC according to standardized protocol (7). Three systolic and diastolic BP measurements were obtained for each participant using a mercury sphygmomanometer. Based on the average of three BP measurements and using methods similar to those previously published (8, 9), we classified participants as having BP in the hypertensive range if BP was ≥95th percentile for age, sex, and height and BP in the pre-hypertensive range if BP was ≥90th percentile and <95th percentile for age, sex, and height. A 60 s pulse [heart rate (HR)] was calculated by multiplying the 30-s resting HR times two (1). Participants receiving antihypertensive medications were excluded from this study.

Body mass index (BMI) was calculated as weight in kilograms divided by height in meters squared (kg/m^2^). BMI *z*-scores were calculated based on 2000 CDC growth charts (10).

Laboratory procedures are described in detail in the *NHANES General Information for Public Files* and *Laboratory Files* (11, 12). High-sensitivity C-reactive protein (CRP) was measured by latex-enhanced nephelometry. Serum cholesterol levels were measured on the Beckman Synchron LX20 (Beckman Coulter, Brea, CA, USA). Urinary albumin levels were measured by using a solid-phase fluorescent immunoassay. Urinary creatinine levels were measured by using the Jaffe rate reaction with a CX3 analyzer (Beckman ASTRA, Brea, CA, USA). The urinary albumin/creatinine ratio (ACR) was calculated as urinary albumin divided by urinary creatinine. An ACR ≥30 was used to define albuminuria. Serum creatinine was measured by means of the modified kinetic Jaffe reaction during the survey period. Because the Jaffe method is known to overestimate eGFR compared to current “gold standard,” glomerular filtration rate (eGFR) was estimated using the new Schwartz formula (13), which has a constant that on average estimates GFR to be lower by 20% compared to the old formula in the adolescent population. Also, the new Schwartz formula is close to the Counahan–Barratt formula used in Europe to estimate GFR.

Attention-deficit hyperactivity disorder was ascertained through self-report among NHANES participants aged 12–18 years of age. Participants were asked whether or not they were ever told by a doctor or health professional that they had attention-deficit disorder.

### Statistical analysis

Continuous, normally distributed variables are presented as mean ± SD and categorical variables are presented as % (*n*). *p*-Values were calculated by weighted least squares or weighted logistic regression analyses. Continuous, abnormally distributed variables are presented as median (25th, 75th percentile). Given that the prevalence and use of medication for ADHD is more common among males, we tested the hypothesis that BP (systolic and diastolic percentile) was influenced by the sex of the ADHD child. An interaction product term of sex × BP percentile (systolic and diastolic separately) was entered into two regression models to represent the interaction. Statistical regression models adjusted for ADHD (with and without CNS stimulant use), age, BMI *z*-score, ethnicity (white vs. non-white), fasting glucose, total cholesterol, eGFR, CRP, HR, and poverty.

Standard errors for all estimates were obtained with the Taylor-linearized variance estimation. Six year sample weights were applied to account for the complex sampling design of NHANES. This includes unequal probabilities of selection, over-sampling, and non-response. All statistical tests used a two-tailed α of <0.05 and were run on Stata (SE 11.2; College Station, TX, USA).

## Results

Table 1 presents the characteristics of the 4,907 children aged 12–18 years in NHANES 1999–2004 meeting the study inclusion criteria. Since this was a representative sample of the US population, these children represent nearly 23 million non-institutionalized US children between the ages of 12–18 years in the US. Among these, 10.8% (*n* = 383) had a self-reported diagnosis of ADHD; 7.3% (*n* = 272) were not on ADHD CNS stimulant medications; and 3.4% (*n* = 111) were on CNS stimulant medications. Mean age was 15 years (SD 2.8). Children with ADHD on CNS stimulant medication were significantly younger, male, and white (*p* < 0.05) compared to those with ADHD not on medication and those without ADHD. HR was significantly higher in children with ADHD on CNS stimulants (81.72 beats/min) compared to children with ADHD and not on stimulants (76.33 beats/min) and children with no ADHD (76.76 beats/min) (Table 1). BMI *z*-score, eGFR, cholesterol, the prevalence of albuminuria, and poverty were not significantly different between the three groups.

**Table 1:** **Demographic and bio-clinical characteristics of study population^a^^–^^c^**.

	ADHD on CNS stimulants	ADHD not on CNS stimulants	No ADHD	Total
	3.41% (*n* = 111)	7.34% (*n* = 272)	89.24% (*n* = 4524)	*n* = 4,907
Age (years)^b^	13.99 (2.07)	14.91 (2.34)	14.99 (2.84)	14.95 (2.79)
Sex (male)^d^	75.93 (85)	72.93 (196)	47.59 (2,163)	50.41 (2,444)
Race^d^				
White	75.38 (49)	75.86 (113)	60.15 (1,101)	61.83 (1263)
Black	7.81 (29)	11.26 (89)	14.34 (1,383)	13.89 (1501)
Mexican-American	5.31 (21)	4.83 (49)	11.49 (1,677)	10.79 (1747)
Other	11.49 (12)	8.05 (21)	14.02 (363)	13.49 (396)
Poverty (yes)	37.34 (47)	47.53 (154)	45.26 (2,696)	45.15 (2,897)
BMI *z*-score	0.32 (1.46)	0.61 (1.49)	0.57 (1.49)	0.53 (1.50)
Height (cm)^c^	162.49 (15.19)	167.84 (12.80)	165.35 (14.34)	165.44 (14.38)
Weight (kg)^c^	58.39 (24.02)	66.77 (24.83)	63.40 (24.60)	63.48 (24.82)
eGFR (ml/min/1.73 m^2^)^b^	109.68 (92.85)	108.96 (95.46)	103.29 (110.84)	103.92 (109.32)
ACR (μg/mg)^b^	7.30 (4.68, 15.44)	6.16 (3.74, 11.65)	6.49 (4.29, 12.59)	6.51 (4.23, 12.59)
CRP (mg/dl)	0.11 (0.26)	0.22 (0.58)	0.16 (0.60)	0.16 (0.59)
Total cholesterol (mg/dl)	156.64 (32.78)	159.54 (37.40)	160.24 (44.17)	160.06 (43.34)
BP classification range				
Normotensive	82.64 (91)	84.19 (228)	85.12 (3,791)	84.96 (4110)
Pre-hypertensive	13.79 (16)	13.74 (37)	12.19 (584)	12.36 (637)
Hypertensive	3.57 (4)	2.06 (7)	2.69 (149)	2.67 (160)
Systolic BP percentile	40.87 (31.93)	37.53 (30.70)	38.46 (37.52)	38.48 (36.89)
Diastolic BP percentile	38.78 (33.94)	38.68 (33.02)	41.54 (37.63)	41.23 (37.28)
Pulse (beats/min)^b^	81.72 (17.03)	76.33 (15.62)	76.76 (17.57)	76.90 (17.54)

*^a^Continuous variables presented as mean ± SD and categorical variables presented as % (*n*). *p*-Values calculated by weighted chi-square analysis or weighted least squares with no ADHD as the reference*.

*^b^*p* < 0.05 for comparison between ADHD on CNS stimulants and no ADHD*.

*^c^*p* < 0.05 for comparison between ADHD not on CNS stimulants and no ADHD*.

*^d^*p* < 0.05 for chi-square analysis between ADHD with and without CNS stimulants and no ADHD*.

Among the study sample, 2.7% (*n* = 160) children had a mean BP in the hypertensive and 12.4% (*n* = 637) in the pre-hypertensive range. The prevalence of abnormal BP (hypertensive and/or pre-hypertensive range) was not significantly different between children with ADHD on CNS stimulant medication compared to children with ADHD without medication and those without ADHD. Systolic and diastolic BP percentiles were also not significantly different between the three groups (Table 1).

In linear regression models with systolic and diastolic BP percentile (BP percentile for age, sex, and height) as the outcome, we tested the hypothesis that BP may be influenced by the sex of the child with ADHD on CNS stimulants. A product interaction term of sex × systolic and diastolic BP percentile was entered into two separate regression models along with the lower level terms. The interaction terms (sex × systolic BP percentile) and (sex × diastolic BP percentile) were statistically significant (*p* < 0.01) in models that included both main effect terms along with ADHD (with and without CNS stimulant use), age, BMI *z*-score, ethnicity (white vs. non-white), fasting glucose, total cholesterol, eGFR, CRP, HR, and poverty.

Table 2 presents the study population characteristics by sex. Females with ADHD and on CNS stimulants were significantly older, shorter in stature, and had a lower ACR than those without ADHD. Conversely, males with ADHD and on CNS stimulants were significantly younger, and had a higher CRP and HR compared to those without ADHD.

**Table 2:** **Demographic and bio-clinical characteristics of study population among (A) females (*n* = 2,463)^a^^–^^c^ and (B) males (*n* = 2,444)^a^^–^^c^**.

	ADHD on CNS stimulants	ADHD not on CNS stimulants	No ADHD	Total
**(A) FEMALES**				
	*n* = 26	*n* = 76	*n* = 2361	*n* = 2,463
Age (years)^b^	14.95 (2.82)	14.83 (2.40)	13.77 (2.23)	14.93 (2.80)
Race				
White	1.19 (12)	2.92 (28)	57.32 (594)	61.62 (634)
Black	0 (3)	0.52 (26)	13.55 (706)	14.13 (735)
Mexican-American	0.15 (8)	0.21 (15)	10.53 (868)	10.90 (891)
Other	0.26 (3)	0.36 (7)	12.94 (193)	13.55 (203)
Poverty (yes)	31.85 (7)	48.21 (44)	46.75 (1417)	46.56 (1468)
BMI *z*-score	0.91 (0.99)	0.70 (1.31)	0.57 (1.41)	0.59 (1.41)
Height (cm)^b^	156.21 (9.24)	162.47 (6.92)	161.00 (9.82)	160.98 (9.91)
Weight (kg)	57.14 (17.58)	63.72 (23.45)	60.24 (21.99)	60.33 (22.07)
eGFR (ml/min/1.73 m^2^)	121.15 (78.45)	87.57 (94.66)	99.37 (106.25)	99.26 (105.51)
ACR (μg/mg)^b^	7.58 (6.43, 15.44)	8.81 (4.70, 12.78)	7.93 (5.14, 15)	7.94 (5.13, 14.82)
CRP (mg/dl)	0.18 (0.35)	0.27 (0.79)	0.17 (0.62)	0.18 (0.62)
Total cholesterol (mg/dl)	158.46 (23.58)	171.05 (47.89)	161.97 (42.57)	162.28 (42.70)
BP classification range^d^				
Normotensive	75.43 (31)	94.13 (100)	91.04 (3039)	90.9 (3170)
Pre-hypertensive	11.45 (2)	5.65 (6)	6.21 (214)	6.27 (222)
Hypertensive	13.11 (3)	0.21 (1)	2.76 (104)	2.83 (108)
Systolic BP percentile	46.92 (34.56)	38.71 (29.99)	37.85 (36.08)	38.03 (35.91)
Diastolic BP percentile	44.47 (34.90)	43.99 (31.78)	44.48 (36.66)	44.46 (36.49)
Pulse (beats/min)	82.17 (17.21)	82.78 (16.50)	79.04 (17.13)	79.24 (0.47)
**(B) MALES**				
	*n* = 85	*n* = 196	*n* = 2163	*n* = 2,444
Age (years)^b^	14.06 (2.01)	14.94 (2.32)	15.03 (2.85)	14.97 (2.77)
Race^d^				
White	76.55 (37)	76.98 (85)	59.49 (507)	62.22 (629)
Black	9.08 (26)	10.64 (63)	14.31 (677)	13.65 (766)
Mexican -American	4.12 (13)	4.66 (34)	11.85 (809)	10.69 (856)
Other	10.25 (9)	7.71 (14)	14.36 (170)	13.44 (193)
Poverty (yes)	39.08 (40)	47.28 (110)	43.61 (1,279)	43.77 (1,429)
BMI *z*-score	0.16 (1.64)	0.58 (1.54)	0.56 (1.58)	0.54 (1.59)
Height (cm)^b^	164.48 (16.04)	169.83 (13.56)	170.15 (15.33)	169.82 (15.39)
Weight (kg)^b^	58.79 (25.76)	67.90 (25.17)	66.88 (26.35)	66.57 (26.51)
eGFR (Schwartz)	106.05 (96.74)	116.89 (93.95)	107.60 (115.43)	108.51 (112.48)
ACR	6.81 (3.97, 16.24)	5.14 (3.55, 11.03)	5.33 (3.65, 9.78)	5.38 (3.65, 10.00)
CRP (mg/dl)^b^	0.90 (0.22)	0.20 (0.48)	0.15 (0.58)	0.15 (0.55)
Total cholesterol (mg/dl)	156.07 (35.25)	155.26 (31.35)	158.33 (45.74)	157.89 (43.73)
BP classification range				
Normotensive	84.92 (111)	80.51 (213)	78.6 (2,526)	79.12 (2,850)
Pre-hypertensive	14.53 (15)	16.75 (41)	18.79 (511)	18.35 (567)
Hypertensive	0.54 (5)	2.75 (8)	2.62 (100)	2.53 (113)
Systolic BP percentile	38.95 (30.68)	37.09 (30.93)	39.14 (39.03)	38.92 (37.80)
Diastolic BP percentile	36.98 (33.36)	36.71 (33.12)	38.31 (38.17)	38.07 (37.51)
Pulse (beats/min)^b^	81.58 (16.97)	73.94 (14.26)	74.25 (17.38)	74.59 (17.25)

*^a^Continuous variables presented as mean ± SD and categorical variables presented as % (*n*). *p*-Values calculated by weighted chi-square analysis or weighted least squares with no ADHD as the reference*.

*^b^*p* < 0.05 for comparison between ADHD on CNS stimulants and no ADHD*.

*^c^*p* < 0.05 for comparison between ADHD not on CNS stimulants and no ADHD*.

*^d^*p* < 0.05 for chi-square analysis between ADHD with and without CNS stimulants and no ADHD*.

Tables 3 and 4 present results for regression analyses with systolic and diastolic BP percentile as the outcome stratified by sex. In each of these models, children with ADHD (with and without the use of CNS stimulants) are compared to those without ADHD (the reference group). For females, only poverty and eGFR are significant predictors of systolic BP percentile while adjusting for other covariates. Among males, age, BMI percentile, ethnicity (white), and total cholesterol are significant predictors of systolic BP percentile while adjusting for other covariates. It is worth noting that the SBP coefficient for females with ADHD on CNS stimulants (*n* = 26) is 2.5 times greater than for males (*n* = 85); 6.76 compared to 2.61.

**Table 3:** **Systolic blood pressure percentile linear regression models by sex**.

Systolic percentile	Coefficient	95% Confidence interval		*p*-value
**FEMALES**				
ADHD not on CNS stimulant	−0.57	−8.23	7.10	0.88
ADHD on CNS stimulant	6.76	−8.33	21.85	0.37
Age (years)	−0.27	−1.07	0.53	0.51
BMI *z*-score	6.64	5.18	8.10	<0.001
White (yes)	−1.42	−4.38	1.54	0.34
Glucose (mg/dl)	0.02	−0.18	0.18	0.99
eGFR (ml/min/1.73 m^2^)	0.02	0.00	0.04	0.03
CRP (mg/dl)	1.63	−1.68	4.93	0.33
Poverty (yes)	3.93	0.75	7.12	0.02
Total cholesterol	−0.01	−0.05	0.04	0.76
Pulse (beats/min)	0.23	0.11	0.35	<0.001
**MALES**				
ADHD not on CNS stimulant	−1.17	−5.49	3.15	0.59
ADHD on CNS stimulant	2.61	−4.40	9.61	0.46
Age (years)	−1.23	−1.89	−0.57	<0.001
BMI *z*-score	7.19	5.81	8.56	<0.001
White (yes)	−5.00	−8.72	−1.30	<0.01
Glucose (mg/dl)	0.10	0.00	0.20	0.05
eGFR (ml/min/1.73 m^2^)	−0.01	−0.03	0.02	0.66
CRP (mg/dl)	−1.30	−4.90	2.30	0.47
Poverty (yes)	−0.08	−3.73	3.56	0.96
Total cholesterol	0.07	0.03	0.12	<0.01
Pulse (beats/min)	−0.01	−0.13	0.12	0.94

**Table 4:** **Diastolic blood pressure percentile linear regression models by sex**.

Diastolic percentile	Coefficient	95% Confidence Interval		*p*-value
**FEMALES**				
ADHD not on CNS stimulant	−0.63	−7.30	6.04	0.85
ADHD on CNS stimulant	0.07	−13.89	14.03	0.99
Age (years)	1.32	0.48	2.15	<0.01
BMI *z*-score	−1.54	−3.01	−0.07	0.04
White (yes)	1.82	−1.21	4.86	0.23
Glucose (mg/dl)	0.05	−0.09	0.19	0.50
eGFR (ml/min/1.73 m^2^)	0.06	0.03	0.08	<0.001
CRP (mg/dl)	−1.46	−3.21	0.30	0.10
Poverty (yes)	2.80	−0.78	6.39	0.12
Total cholesterol	0.02	−0.03	0.07	0.42
Pulse (beats/min)	0.24	0.13	0.35	<0.001
**MALES**				
ADHD not on CNS stimulant	−1.84	−7.53	3.85	0.52
ADHD on CNS stimulant	−4.03	−9.94	1.87	0.18
Age (years)	0.05	−0.80	0.90	0.91
BMI *z*-score	−2.35	−3.57	−1.13	<0.001
White (yes)	−0.36	−3.21	2.48	0.80
Glucose (mg/dl)	0.01	−0.09	0.11	0.86
eGFR (ml/min/1.73 m^2^)	0.06	0.03	0.08	<0.001
CRP (mg/dl)	−1.07	−5.05	2.91	0.59
Poverty (yes)	−0.16	−3.07	2.75	0.91
Total cholesterol	0.02	−0.03	0.06	0.47
Pulse (beats/min)	0.27	0.11	0.42	<0.01

For both males and females BMI *z*-score, eGFR, and HR were significant predictors of diastolic BP percentile while adjusting for other covariates. Among females only age was a significant predictor of diastolic BP percentile.

## Discussion

To our knowledge, this is the largest study to examine the effect of CNS stimulant medication use on BP classification in a study population representative of the US children and adolescents. In this study, we report comparable prevalence of elevated BP (pre-hypertensive and hypertensive range) between patients with self-reported ADHD vs. no ADHD diagnosis. Also, we report comparable prevalence of elevated BP between the ADHD patients on stimulant medications vs. no medications. HR was significantly higher in the group on CNS stimulant medications compared to children with ADHD on no stimulants and no ADHD.

It is important to point out that children with self-reported ADHD on stimulant medications had a slightly higher systolic BP percentile, which was not significantly different between the groups and did not change BP classification. We also report a significant difference in BP classification by sex; females with ADHD are significantly more hypertensive, while males with ADHD on CNS stimulants had a significantly higher HR.

Ten percent of this nationally representative sample of 4,907 children had ADHD. One-third of children with ADHD were on medications, 90% of whom were on CNS stimulants. Methylphenidate and amphetamine–dextroamphetamine were the two most commonly used medications.

Our findings are consistent with a meta-analysis of adult studies examining the relative change in various CV parameters associated with ADHD treatment modalities, Mick et al. reported that subjects randomized to CNS stimulant treatment demonstrated a statistically significant increased resting HR [+5.7 bpm (95% CI: 3.6, 7.8), *p* < 0.001] and systolic BP findings [+2.0 mmHg (95% CI: 0.8, 3.2), *p* = 0.005] compared with subjects randomized to placebo (14). Samuels and colleagues examined the effect of stimulants on 24-h ambulatory BP in children with ADHD, in a double-blind, randomized, cross-over trial they reported elevations in most hemodynamic parameters derived from ABPM during the active treatment period; overall diastolic BP (69.7 vs. 65.8 mmHg, *p* = 0.02) and waking diastolic BP (75.5 vs. 72.3 mmHg, *p* = 0.03) were significantly higher during active treatment. Total HR was also significantly higher during active treatment (85.5 vs. 79.9 beats/min, *p* = 0.004) (15). In a systematic review by Westover and colleagues examining the relationship between stimulant medications and CV events, 10 population-based observational studies were reviewed. Six out of seven studies in children and adolescents did not show an association between stimulant use and adverse CV outcomes. In contrast, two out of three studies in adults found an association. The authors concluded that findings of an association between prescription stimulant use and adverse CV outcomes are mixed. Studies of children and adolescents suggest that statistical power is limited in available study populations, and the absolute risk of an event is low (16).

The duration of CNS stimulant medication effects on CV parameters is not well understood, it is possible that these effects are less prominent with time. Hammerness and colleagues examined the effects of high doses of extended-release methylphenidate (OROS MPH) on CV variables in 114 adolescents with ADHD, they reported small but statistically significant changes in DBP and HR at 6 weeks, without further increases up to 6 months follow-up. A small but not statistically significant increase in SBP was observed at 6 weeks, this increase was statistically significant at 6 months of follow-up (17). It is important to point out that male and female participant data were combined and analyzed together in this study, the study did not classify BP or present BP percentiles and did not comment on change in BP classification with increased BP. On the other hand, this study demonstrated a significant increase in HR in the first 6 weeks, which was sustained until the end of the follow-up period; one patient had to stop the treatment due to intermittent palpitations. Among the 1758 youth enrolled in the Italian ADHD National Registry, statistically significant increases were observed in CV measures: in the methylphenidate group after 6 months in HR (+2.01, *p* = 0.01); in the atomoxetine group after 6 months in diastolic pressure (+1.60, *p* = 0.01) and in HR (+2.93, *p* = 0.001), and after 12 months in HR (+3.26, *p* = 0.003) (18).

In adults, high HR has proven to be a strong predictor of CV disease and a predictor of the development of essential HTN. Whether HR itself is a risk factor for development of HTN or just a marker for sympathetic over activation is still a matter of debate (19, 20).

Previous studies showed a small increase in HR and BP measurements. It is important to point out that while children on CNS stimulant treatment in our study had similar effects, those small changes in BP measurements did not change BP classification range between the different groups. On the other hand, it is important to remember that the long-term CV effects of elevated resting HR and the small increases in BP are not well defined in children and may contribute to an increased future CV risk. Using two large administrative databases, Schelleman and colleagues compared the rate of severe CV events and death in children who use attention-deficit/hyperactivity disorder (ADHD) medications vs. non-users. They found the rate of CV events in exposed children to be very low and in general no higher than that in unexposed control subjects. Because of the low number of events, they had limited ability to rule out relative increases in event rate (11).

We found males with ADHD on CNS stimulants had a lower BMI and significantly higher CRP levels. While it is well described that treatment with CNS stimulants can depress growth in hyperactive children on stimulant drugs (21), the relationship between CRP levels and CNS stimulant medication treatment is less well understood. Researchers have described a significant association between development of Raynaud’s syndrome and therapy with CNS stimulants used for the treatment of ADHD in a population of children followed at the rheumatology clinic. In that patient population, both CRP and ESR were elevated. The authors attributed the findings to underlying inflammatory disease (22). Others have reported methylphenidate and dextroamphetamine to induce peripheral vasculopathy (23, 24).

The relationship between BP and GFR is complicated. In our study, higher GFR was an independent determinant of systolic BP in females and diastolic BP in both males and females (Tables 3 and 4). The underlying mechanisms behind this relationship are unclear, but someone can speculate on possible hemodynamic changes that may affect the renal blood flow and the glomerular filtration pressure. With the help of renal auto regulation, GFR is maintained relatively stable over a wide range of BP in healthy individuals; it is unclear how CNS stimulants and increased sympathetic outflow affect this relationship.

Our study is limited by its cross-sectional design and the inability to draw any causal relationships. All three BP measures were taken at one sitting 5 min apart. As such, none of the children included in the study can be diagnosed truly hypertensive or pre-hypertensive based on the fourth report recommendations. Rather, the average of three BP measures here serve as a proxy for BP status similar to many other published studies (8, 9). Another limitation of the study is our use of the modified Schwartz formula to estimate GFR, while the formula has not been validated in healthy children and adolescents, a modified formula uses a constant that yields an eGFR that is lower by almost 20–30% compared to the original formula, this allowed correcting for the use of the Jaffe method to measure serum creatinine that was used during the survey period. Although patients were queried about medication usage, being a cross-sectional study, we neither know the duration of CNS stimulant use nor do we know compliance of use. There is also a possibility that study subjects have not answered questions honestly with respect to diagnosis of ADHD. However, since NHANES is not a survey about ADHD, there should be no reason for an individual to bias results and not be truthful about this history. Nonetheless, there is a possibility that some may have over- or under-reported. Similarly, some may have over- or under-reported taking medication for ADHD. However, NHAHES asks that survey participants bring all medication to the mobile examination and examiners record the medications directly from the containers brought in from the patient. This would reduce the likelihood of bias. It is also possible that a child was on a medication break and this was not reported. After stratifying by sex, the number of children on CNS stimulant medications with BP in the hypertensive and pre-hypertensive range was relatively small.

On the other hand, this study has a number of strengths in that it examines a large pediatric cohort, representative of the US population. Also, in this study, we *classified* each patient’s BP based on the average of three measurements obtained during the survey exam and adjusted for height and sex: normal BP, hypertensive, or pre-hypertensive range. In this study, we were able to compare a subgroup of self-reported ADHD patients on CNS stimulants to a larger group of self-reported ADHD patients on no medications as well as to healthy counterparts.

## Conclusion

Overall, children on CNS stimulants tend to have higher HRs and slightly higher BP percentiles. The difference in BP percentiles did not translate into a difference in the prevalence of BP in the hypertensive and/or pre-hypertensive ranges between children with ADHD on stimulant treatment vs. no treatment and was comparable to children without ADHD. Sex differences in BP classification may exist in children on CNS stimulant treatment. These findings as well as the higher CRP levels in males on CNS stimulants need to be confirmed by future studies. Prescribers of CNS stimulant medication need to be aware of the potential long-term adverse effects of these medications and need to weigh the risk and benefits when starting stimulant treatment.

## Author Contributions

Authors (Susan M. Hailpern, Brent M. Egan, Carol Wagner, Kimberly D. Lewis, Ghassan F. Shattat, Doaa I. Al Qaoud, and Ibrahim F. Shatat) had substantial contributions to the conception or design of the work; or the acquisition, analysis, or interpretation of data for the work; and all authors contributed to drafting the work or revising it critically for important intellectual content; and the final version was approved by all authors. All authors agree to accountable for all aspects of the work in ensuring that questions related to the accuracy or integrity of any part of the work are appropriately investigated and resolved.

## Conflict of Interest Statement

The authors declare that the research was conducted in the absence of any commercial or financial relationships that could be construed as a potential conflict of interest.

## References

[1] National High Blood Pressure Education Program Working Group on High Blood Pressure in Children and Adolescents. The fourth report on the diagnosis, evaluation, and treatment of high blood pressure in children and adolescents. Pediatrics (2004) 114(2 Suppl 4th Report):555–76 10.1542/peds.114.2.S2.555 15286277

[2] Karpettas N Nasothimiou E Kollias A Vazeou A Stergiou GS Ambulatory and home blood pressure monitoring in children and adolescents: diagnosis of hypertension and assessment of target-organ damage. Hypertens Res (2013) 36(4):285–92 10.1038/hr.2012.220 23344131

[3] Berenson GS Srinivasan SR Bao W Newman WP III Tracy RE Wattigney WA Association between multiple cardiovascular risk factors and atherosclerosis in children and young adults. The Bogalusa Heart Study. N Engl J Med (1998) 338(23):1650–6 10.1056/NEJM199806043382302 9614255

[4] Lande MB Kaczorowski JM Auinger P Schwartz GJ Weitzman M Elevated blood pressure and decreased cognitive function among school-age children and adolescents in the United States. J Pediatr (2003) 143(6):720–4 10.1067/S0022-3476(03)00412-8 14657815

[5] Centers for Disease Control and Prevention (CDC). Increasing prevalence of parent-reported attention-deficit/hyperactivity disorder among children – United States, 2003 and 2007. MMWR Morb Mortal Wkly Rep (2010) 59(44):1439–43 21063274

[6] Nissen SE ADHD drugs and cardiovascular risk. N Engl J Med (2006) 354(14):1445–8 10.1056/NEJMp068049 16549404

[7] National Health and Nutrition Examination Survey: Physician Examination Procedures Manual. Atlanta: Centers for Disease Control and Prevention (2003). Available from: http://www.cdc.gov/nchs/data/nhanes/nhanes_03_04/PE.pdf

[8] Muntner P He J Cutler JA Wildman RP Whelton PK Trends in blood pressure among children and adolescents. JAMA (2004) 291(17):2107–13 10.1001/jama.291.17.2107 15126439

[9] Rosner B Cook NR Daniels S Falkner B Childhood blood pressure trends and risk factors for high blood pressure: the NHANES experience 1988-2008. Hypertension (2013) 62(2):247–54 10.1161/HYPERTENSIONAHA.113.02128 23856492 PMC3769135

[10] Kuczmarski RJ Ogden CL Guo SS Grummer-Strawn LM Flegal KM Mei Z 2000 CDC growth charts for the United States: methods and development. Vital Health Stat 11 (2002) 246:1–190 12043359

[11] Schelleman H Bilker WB Strom BL Kimmel SE Newcomb C Guevara JP Cardiovascular events and death in children exposed and unexposed to ADHD agents. Pediatrics (2011) 127(6):1102–10 10.1542/peds.2010-3371 21576311 PMC3387871

[12] National Health and Nutrition Examination Survey: General Information for the Public Files of the 2007-2008 Laboratory Data. Atlanta: Centers for Disease Control and Prevention (2010). Available from: http://www.cdc.gov/nchs/nhanes/nhanes2007-2008/labdoc_e.htm

[13] Schwartz GJ Munoz A Schneider MF Mak RH Kaskel F Warady BA New equations to estimate GFR in children with CKD. J Am Soc Nephrol (2009) 20(3):629–37 10.1681/ASN.2008030287 19158356 PMC2653687

[14] Mick E McManus DD Goldberg RJ Meta-analysis of increased heart rate and blood pressure associated with CNS stimulant treatment of ADHD in adults. Eur Neuropsychopharmacol (2013) 23(6):534–41 10.1016/j.euroneuro.2012.06.011 22796229 PMC3488604

[15] Samuels JA Franco K Wan F Sorof JM Effect of stimulants on 24-h ambulatory blood pressure in children with ADHD: a double-blind, randomized, cross-over trial. Pediatr Nephrol (2006) 21(1):92–5 10.1007/s00467-005-2051-1 16254730

[16] Westover AN Halm EA Do prescription stimulants increase the risk of adverse cardiovascular events? A systematic review. BMC Cardiovasc Disord (2012) 12:41 10.1186/1471-2261-12-41 22682429 PMC3405448

[17] Hammerness P Wilens T Mick E Spencer T Doyle R McCreary M Cardiovascular effects of longer-term, high-dose OROS methylphenidate in adolescents with attention deficit hyperactivity disorder. J Pediatr (2009) 155(1):84–9 10.1016/j.jpeds.2009.02.008 19394037

[18] Arcieri R Germinario EA Bonati M Masi G Zuddas A Vella S Cardiovascular measures in children and adolescents with attention-deficit/hyperactivity disorder who are new users of methylphenidate and atomoxetine. J Child Adolesc Psychopharmacol (2012) 22(6):423–31 10.1089/cap.2012.0014 23362511

[19] Tjugen TB Flaa A Kjeldsen SE High heart rate as predictor of essential hypertension: the hyperkinetic state, evidence of prediction of hypertension, and hemodynamic transition to full hypertension. Prog Cardiovasc Dis (2009) 52(1):20–5 10.1016/j.pcad.2009.05.008 19615489

[20] Tjugen TB Flaa A Kjeldsen SE The prognostic significance of heart rate for cardiovascular disease and hypertension. Curr Hypertens Rep (2010) 12(3):162–9 10.1007/s11906-010-0104-8 20431967

[21] Safer D Allen R Barr E Depression of growth in hyperactive children on stimulant drugs. N Engl J Med (1972) 287(5):217–20 10.1056/NEJM197208032870503 4556640

[22] Goldman W Seltzer R Reuman P Association between treatment with central nervous system stimulants and Raynaud’s syndrome in children: a retrospective case-control study of rheumatology patients. Arthritis Rheum (2008) 58(2):563–6 10.1002/art.23301 18240233

[23] Syed RH Moore TL Methylphenidate and dextroamphetamine-induced peripheral vasculopathy. J Clin Rheumatol (2008) 14(1):30–3 10.1097/RHU.0b013e3181639aaa 18431096

[24] Yu ZJ Parker-Kotler C Tran K Weller RA Weller EB Peripheral vasculopathy associated with psychostimulant treatment in children with attention-deficit/hyperactivity disorder. Curr Psychiatry Rep (2010) 12(2):111–5 10.1007/s11920-010-0093-y 20425295

